# DeepWalk based method to predict lncRNA-miRNA associations via lncRNA-miRNA-disease-protein-drug graph

**DOI:** 10.1186/s12859-022-04579-0

**Published:** 2022-02-25

**Authors:** Long Yang, Li-Ping Li, Hai-Cheng Yi

**Affiliations:** 1 Xinjiang Technical Institute of Physics and Chemistry, Chinese Academy of Sciences, Urumqi, 830011 China; 2 University of Chinese Academy of Sciences, Beijing, 100049 China; 3 College of Grassland and Environmental Science, Xinjiang Agricultural University, Urumqi, 830052 China

**Keywords:** DeepWalk, LncRNA, miRNA, Random forest

## Abstract

**Background:**

Long non-coding RNAs (lncRNAs) play a crucial role in diverse biological processes and have been confirmed to be concerned with various diseases. Largely uncharacterized of the physiological role and functions of lncRNA remains. MicroRNAs (miRNAs), which are usually 20–24 nucleotides, have several critical regulatory parts in cells. LncRNA can be regarded as a sponge to adsorb miRNA and indirectly regulate transcription and translation. Thus, the identification of lncRNA-miRNA associations is essential and valuable.

**Results:**

In our work, we present DWLMI to infer the potential associations between lncRNAs and miRNAs by representing them as vectors via a lncRNA-miRNA-disease-protein-drug graph. Specifically, DeepWalk can be used to learn the behavior representation of vertices. The methods of fingerprint, *k*-mer and MeSH descriptors were mainly used to learn the attribute representation of vertices. By combining the above two kinds of information, unknown lncRNA-miRNA associations can be predicted by the random forest classifier. Under the five-fold cross-validation, the proposed DWLMI model obtained an average prediction accuracy of 95.22% with a sensitivity of 94.35% at the AUC of 98.56%.

**Conclusions:**

The experimental results demonstrated that DWLMI can effectively predict the potential lncRNA-miRNA associated pairs, and the results can provide a new insight for related non-coding RNA researchers in the field of combing biology big data with deep learning.

## Background

MicroRNAs (miRNAs) are a wide range of endogenous small non-coding RNA molecules, regulating the expression of target genes via translational inhibition [1–4]. The long non-coding RNAs (lncRNAs) have been shown to involve miscellaneous cellular processes such as protein scaffolding and cell differentiation [5–8]. Recently, there is more and more evidence to show that lncRNA can function as miRNA sponge to participate in various biological processes, besides that, miRNA can lead to a lower effect on mRNAs in the regulation and has an essential role in the molecular level to lncRNA [8–12]. Furthermore, the regulatory networks of lncRNA-miRNA associations can be concerned with pathological processes involved in many diseases, and the potential to use this knowledge to achieve the goal of "precision" or "personalized medicine" in oncology is also discussed [13–19]. Therefore, on the molecular level, the potential associations between lncRNAs and miRNAs in important cell activities can be predicted. Moreover, it is critical and urgent to identify uncovered lncRNA-miRNA associations to facilitate understanding the mechanisms [20–22].

To date, there are three categories of methods for predicting potential lncRNA-miRNA associations. The first kind of method predicts the associations between lncRNA-miRNA by designing the traditional wet experiment. Amanda et al. [23] designed their experiment based on the crosslinking and real-time PCR (RT-qPCR), the experiment results revealed that H19 identify as an important regulator of the major let-7 family of microRNAs. Li et al. [24] using the algorithm of MIRANDA and TARGETSCAN to investigate lncRNA-miRNA associations on a genomic scale. Traditional wet methods are time-consuming and labor-intensive. Benefiting from the high-throughput technologies, many computational methods were used to predict associations between lncRNA-miRNA. These methods are roughly classified as collection methods and prediction methods, collection methods based on the technology of text mining and data analysis, Li et al*.* [25] systematically identify the lncRNA–miRNA associations networks and other related information from 108 CLIP-Seq (PAR-CLIP, HITS-CLIP, iCLIP, CLASH) data sets. Gong et al*.* [26] collected 8091 associations verified by wet experiments between lncRNA-miRNA on account of the SNP experiments required. The limitations of collection methods are obvious, only associations between lncRNA-miRNA proved by wet experiments can be collected. Thus, prediction methods were proposed, in the beginning, prediction methods based on several features of lncRNAs and miRNAs, such as putative functions, expression profiles, and sequences information. For instance, Huang et al. [27] proposed a new way named EPLMI to predict lncRNA-miRNA associations by using the features extracted from expression profiles to represent a bipartite graph of known interactions to construct a prediction model. With the development of technology, some deep learning methods were applied in the field of predicting lncRNA-miRNA associations. Veneziano et al*.* [28] provide a brief update on the actual biomedical relevance of lncRNAs and miRNAs. Wang et al. [29] proposed the GNMFLMI calculation model to construct affinity graphs by p-nearest neighbors. Zhou et al. [30] proposed a method named GEEL based on graph embedding methods to represent latent representations of their network. Zhang et al. [31] proposed a method named SLNPM based linear neighborhood propagation to predict lncRNA-miRNA associations. These models are based on the information of the research objects to detect unknown associations. Computational methods could predict lncRNA-miRNA associations in a short time and provide a novel perspective for predicting other associations [32–36]. Recently, researchers are gradually addressing their research through an increasingly overall perspective. Guo et al. [37] presented the concept of molecular associations to explore potential associations among different biological objects. Ma et al. [38] proposed GABN model to find an optimal alignment between proteins across species. Hu et al. [39] used diverse heterogeneous datasets to explore potential associations.

In this study, we proposed a method DWLMI to predict the associations between lncRNA-miRNA. An original lncRNA-miRNA-disease-protein-drug network was constructed by integrating the attributes information and behavior information of these biological objects, then, we can predict potential lncRNA-miRNA associations through the random forest classifier. Finally, for evaluating the prediction of our model, fivefold cross validation was implemented for DWLMI. As a result, DWLMI obtained substantial performance with the AUC of 98.56% under fivefold cross validation. Moreover, the classifier and method comparison experiment were also applied to evaluate our method from different aspects. To further assess our model, case studies were carried out to verify the ability of our model. This paper makes the following contributions: (1) the experiment offers a new perspective for exploring the associations between lncRNAs and miRNAs through some intermediary; (2) the proposed DWLMI models can effectively predict the potential lncRNA-miRNA associated pairs.

## Result

### Evaluation metrics

To further evaluate the performance of our model, a series of evaluation metrics were used. Cross-validation was used to assess the performance of our evaluation task fairly and comprehensively. In our work, the fivefold cross-validation was chosen to divide the whole data set into five mutually exclusive subsets of equal size, each subset can be regarded as the test set to evaluate our model in turn, the remaining subsets are used to train the model as the training set. After the fivefold cross-validation is implemented, ROC (Receiver Operating Characteristic) curve and PR (Precision-Recall) curve are drawn and calculated separately to evaluate the performance of our model. Generally, the ROC curve is used to evaluate the classifier to show the performance and measure the non-equilibrium in classification tasks. The calculation results of FRP (false positive rate) and TPR (true positive rate) are used to construct the ROC curve, the FPR construct the abscissa of the ROC curve, and the TPR construct the ordinate of the ROC curve. The value of AUC generally ranging from 0.5 to 1. The calculation of AUC is calculating the areas under the ROC curve. The PR curve is also used to evaluate the classification ability of models, specially, PR curve can find more information while dealing with some imbalanced data sets. The areas under the PR curve can be defined as AUPR. Besides that, the extensively used evaluation metrics are used to assess our model including accuracy (Acc.), sensitivity (Sen.), specificity (Spec.), precision (Prec.), and MCC. These evaluation metrics are defined as:

1 $$Acc. = \frac{TN + TP}{{TN + TP + FN + FP}}$$

2 $$Sen. = \frac{TP}{{TP + FN}}$$

3 $$Spec. = \frac{TN}{{TN + FP}}$$

4 $$Prec. = \frac{TP}{{TP + FP }}$$

5 $$MCC = \frac{TP \times TN - FP \times FN}{{\sqrt {\left( {TP + FP} \right)\left( {TP + FN} \right)\left( {TN + FP} \right)\left( {TN + FN} \right)} }}$$

where TN indicates the number of true negative; FN represents the number of false negative; TP stands for the true positive number; FP denoted the false positive number.

### LncRNA-MiRNA associations prediction capability evaluation

For evaluating our model, the known associations between lncRNAs and miRNAs were selected as a complimentary sample, and the same amounts of negative samples were chosen randomly. The training set contains two kinds of samples. To measure the performance of DWLMI that predicts the association of lncRNA-miRNA, we performed fivefold cross-validation to randomly divided the entire data into five parts in equal size. One subset is used as the test set, and others were used as training sets to test the classifier. Then, for each cross-validation, only 80% of the total edges in the current training set would be embedded as the manner of the node. Although the above operations may cause some problems, simulating the real environment for researchers still very dominant through manual experiments is apparent.

**Fig. 1:**
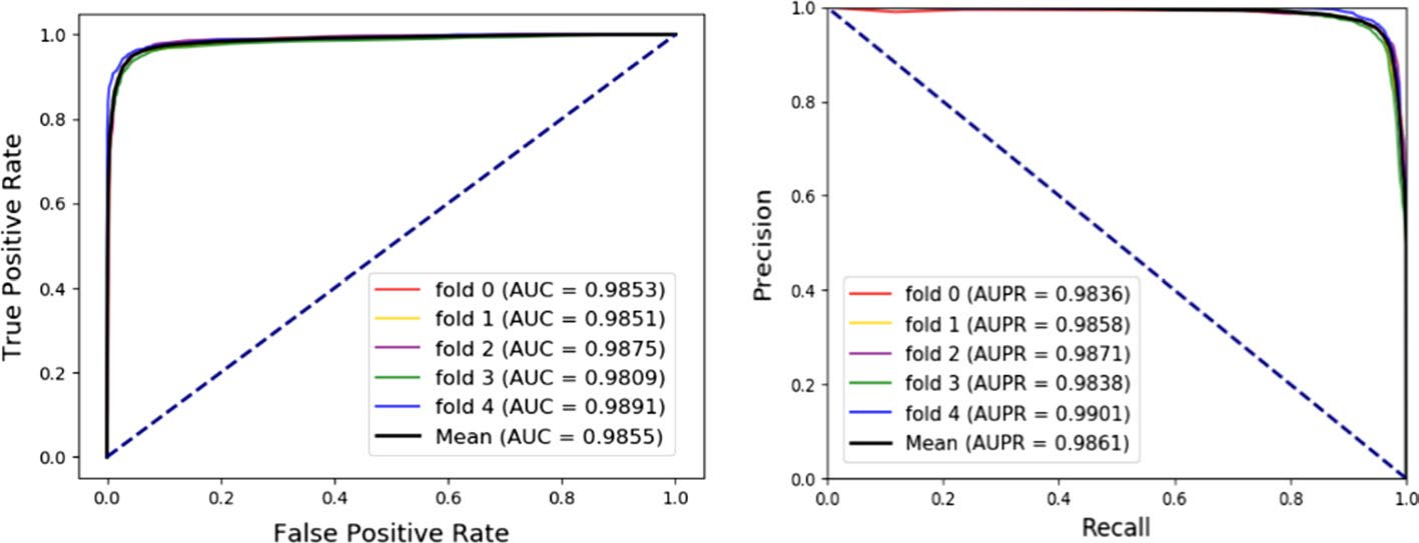
The AUCs, ROCs, AUPRs, and PRs of DWLMI under fivefold cross-validation

As shown in Table 1, the results of average Acc., Sen., Spec., Prec., MCC, and AUC were 95.22%, 94.35%, 96.1%, 96.03%, 90.46%, and 98.56%, respectively, when DWLMI was applied to predict the associations of lncRNA-miRNA. For a better understanding, the ROC curve and the PR curve were also used to evaluate DELMI. ROC curve and PR curve were used to assess our model from a different angle. Our method obtained an AUC of 0.9856, and the results indicated that our method could identify the associations of lncRNA-miRNA effectively (Fig. 1).

**Table 1:** Various evaluation metrics under fivefold cross validation achieved by DWLMI

Fold	Acc. (%)	Sen. (%)	Spec. (%)	Prec. (%)	MCC (%)	AUC (%)
0	95.31	94.81	95.82	95.78	90.63	98.53
1	95.19	94.09	96.3	96.21	90.41	98.51
2	95.31	94.51	96.12	96.06	90.64	98.75
3	94.51	93.19	95.82	95.71	89.05	98.09
4	95.79	95.16	96.42	96.37	91.58	98.91
Average	95.22 ± 0.46	94.35 ± 0.76	96.1 ± 0.27	96.03 ± 0.28	90.46 ± 0.91	98.56 ± 0.31

### Measure our method against feature extraction methods

**Fig. 2:**
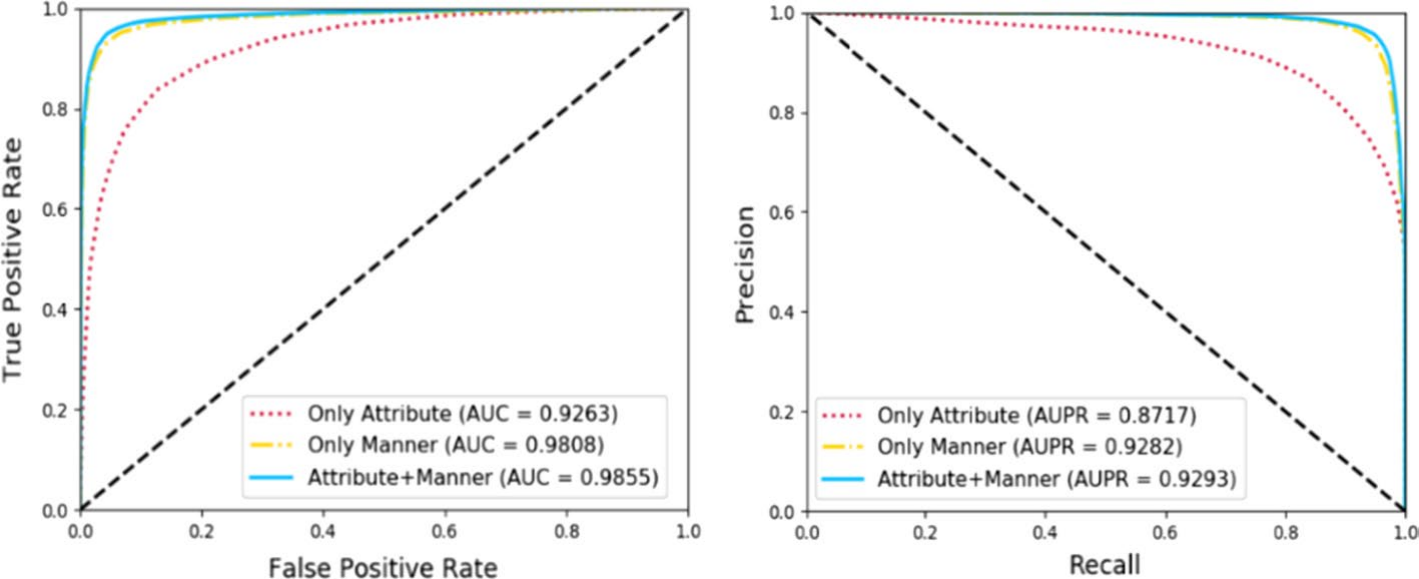
Comparison with different features under fivefold cross-validation

For measuring the effectiveness of our method, respectively, we utilized the attributes information and the behavior information to compare with our method in the extensive evaluation metrics. As presented in Table 2 and Fig. 2, the results of average Acc., Sen., Spec., Prec., MCC, and AUC of 95.22%, 94.35%, 96.1%, 96.03%, 90.46%, and 98.56% demonstrate that the performance of node behavior information with node attribute information can obtain better performance than other feature extraction methods.

**Table 2:** Measuring our method with different features

Feature	Acc. (%)	Sen. (%)	Spec. (%)	Prec. (%)	MCC (%)	AUC (%)
Attribute	85.58 ± 0.64	83.53 ± 0.36	87.63 ± 1.0	87.11 ± 0.95	71.22 ± 1.3	92.63 ± 0.56
Manner	94.33 ± 0.38	92.45 ± 0.77	96.22 ± 0.38	96.07 ± 0.37	88.73 ± 0.74	98.09 ± 0.16
Both	95.22 ± 0.46	94.35 ± 0.76	96.1 ± 0.27	96.03 ± 0.28	90.46 ± 0.91	98.56 ± 0.31

### Compared with other state-of-the-art methods

To further demonstrate the prediction performance of DWLMI, we compared DWLMI with other state-of-the-art methods. Among them, EPLMI [27] predicts lncRNA-miRNA associations by representing known interactions as a bipartite graph. The GNMFLMI [29]calculation model constructs affinity graphs by p-nearest neighbors. GEEL [30] fully exploring the structure of lncRNA-miRNA associations network, using graph embedding methods to represent the heterogeneous network. SLNPM [31] based linear neighborhood propagation to predict lncRNA-miRNA associations. To further evaluate the performance of our model. The AUC values comparison between DWLMI and other state-of-art methods were making, the results are shown in Table 3. The experiment results indicate that DWLMI can effectively predict lncRNA-miRNA associations.

**Table 3:** The AUC values of DWLMI compared with other state-of-art methods

Methods	EPLMI	GNMFLMI	SLNPM	GEEL	DWLMI
AUC	0.8447	0.8894	0.9165	0.9537	0.9856

### Case studies

In our work, case studies are carried out to further verify the ability of DWLMI on predicting potential lncRNA-miRNA associations, we implemented DWLMI on nonhsat159246.1 and hsa-mir-544a as case studies, all associations collected from lncRNASNP2 database. After dealing with the dataset, such as de-redundancy, simplification, and deletion of the irrelevant items, we obtained 466 kinds of lncRNAs and 254 kinds of miRNAs. For nonhsat159246.1, all miRNAs which connect with nonhsat159246.1 were removed from the lncRNASNP2 dataset, after removing the associations from our dataset, the number of positive samples is 8265. Negative samples have the same amounts of positive samples and were randomly selected from disconnect associations as mentioned above. The model of DWLMI is trained by the train sets that consist of positive samples and negative samples. Then, the final prediction results were sorted in descending order according to the prediction score. Table 4 shows the top 20 predicted interactions for this lncRNA.18 out of the top 20 candidate miRNAs are confirmed by the lncRNASNP2 database. The same way is used for hsa-mir-544a, it is worth noting that the number of positive samples was 8303. the prediction results are shown in Table 5, the top 20 predicted miRNAs by DWLMI were verified by the lncRNASNP2 database. The experiment results show that DWLMI can effectively predict lncRNA-miRNA associations.

**Table 4:** The top 20 predicted MiRNAs by DWLMI for nonhsat159246.1 on the lncRNASNP2 dataset

Rank	MiRNAs	Evidences	Rank	MiRNAs	Evidences
1	hsa-mir-455-5p	lncRNASNP2	11	hsa-mir-29a-3p	Unconfirmed
2	hsa-mir-23a-3p	lncRNASNP2	12	hsa-mir-873-5p	lncRNASNP2
3	hsa-mir-23b-3p	lncRNASNP2	13	hsa-mir-3167	lncRNASNP2
4	hsa-mir-23c	lncRNASNP2	14	hsa-mir-221-3p	lncRNASNP2
5	hsa-mir-205-5p	lncRNASNP2	15	hsa-mir-19b-3p	Unconfirmed
6	hsa-mir-544a	lncRNASNP2	16	hsa-mir-221-3p	Unconfirmed
7	hsa-mir-374b-5p	lncRNASNP2	17	hsa-mir-4465	lncRNASNP2
8	hsa-mir-135b-5p	lncRNASNP2	18	hsa-mir-196b-5p	lncRNASNP2
9	hsa-mir-590-3p	lncRNASNP2	19	hsa-mir-29c-3p	lncRNASNP2
10	hsa-mir-29b-3p	unconfirmed	20	hsa-mir-346	lncRNASNP2

**Table 5:** The top 20 predicted LncRNAs by DWLMI for hsa-mir-544a on the lncRNASNP2 dataset

Rank	LncRNAs	Evidences	Rank	LncRNAs	Evidences
1	nonhsat137542.2	lncRNASNP2	11	nonhsat022125.2	lncRNASNP2
2	nonhsat137558.2	lncRNASNP2	12	nonhsat159244.1	lncRNASNP2
3	nonhsat137541.2	lncRNASNP2	13	nonhsat159242.1	lncRNASNP2
4	nonhsat007662.2	lncRNASNP2	14	nonhsat022145.2	lncRNASNP2
5	nonhsat022132.2	lncRNASNP2	15	nonhsat017523.2	Unconfirmed
6	nonhsat159248.1	lncRNASNP2	16	nonhsat007668.2	lncRNASNP2
7	nonhsat159252.1	lncRNASNP2	17	nonhsat007695.2	lncRNASNP2
8	nonhsat159243.1	lncRNASNP2	18	nonhsat026096.2	Unconfirmed
9	nonhsat034665.2	unconfirmed	19	nonhsat007699.2	lncRNASNP2
10	nonhsat035663.2	unconfirmed	20	nonhsat007681.2	lncRNASNP2

## Discussion

In this article, we proposed a new model named DELMI. This model integrates multi-source biological data, besides that, biological entities were represented in heterogeneous attribute networks in a multi-view and multi-modal way. Even in the field of bioinformatics field, technology has made great progress, but there are few tools to integrate multi-source information. The method proposed in this paper is data-driven and represents a preliminary exploration of the combination of computer technology and biological big data. The results demonstrate that our research helps understand various cellular and molecular mechanisms. However, there are still some limitations. For example, we use a very simple representation for the sequence of biological entities and chemical structure, and the prediction results have not been verified by wet experiments. These will be further investigated in future work.

## Conclusions

The associations between lncRNAs and miRNAs have been confirmed to be closely related to diverse biological processes including the development of various diseases. Identifying lncRNA-miRNA associations will be useful for researchers to understand the mechanisms of various diseases. In recent years, accumulating evidence demonstrates the effectiveness of deep learning strategy in big biological. In this article, we proposed a machine learning model based on different molecular relationships and network embedding to detect potential lncRNA-miRNA associations. In our method, each node can be transformed into a vector-based feature of the attributes and embeddings. Then, based on the above information, a 128-dimensional vector can be used to represent every node to train the classifier to predict the lncRNA-miRNA associations. The experimental results suggest that DWLMI can effectively predict the potential lncRNA-miRNA associated pairs. This provides a new insight for related non-coding RNA researchers. There are several deficiencies with our method. For example, we used a very simple representation for the sequence of biological entities and chemical structure, and the prediction results have not been verified by wet experiments. These will be further investigated in future work, and new methods will be used to deal with these problems.

## Methods

**Fig. 3:**
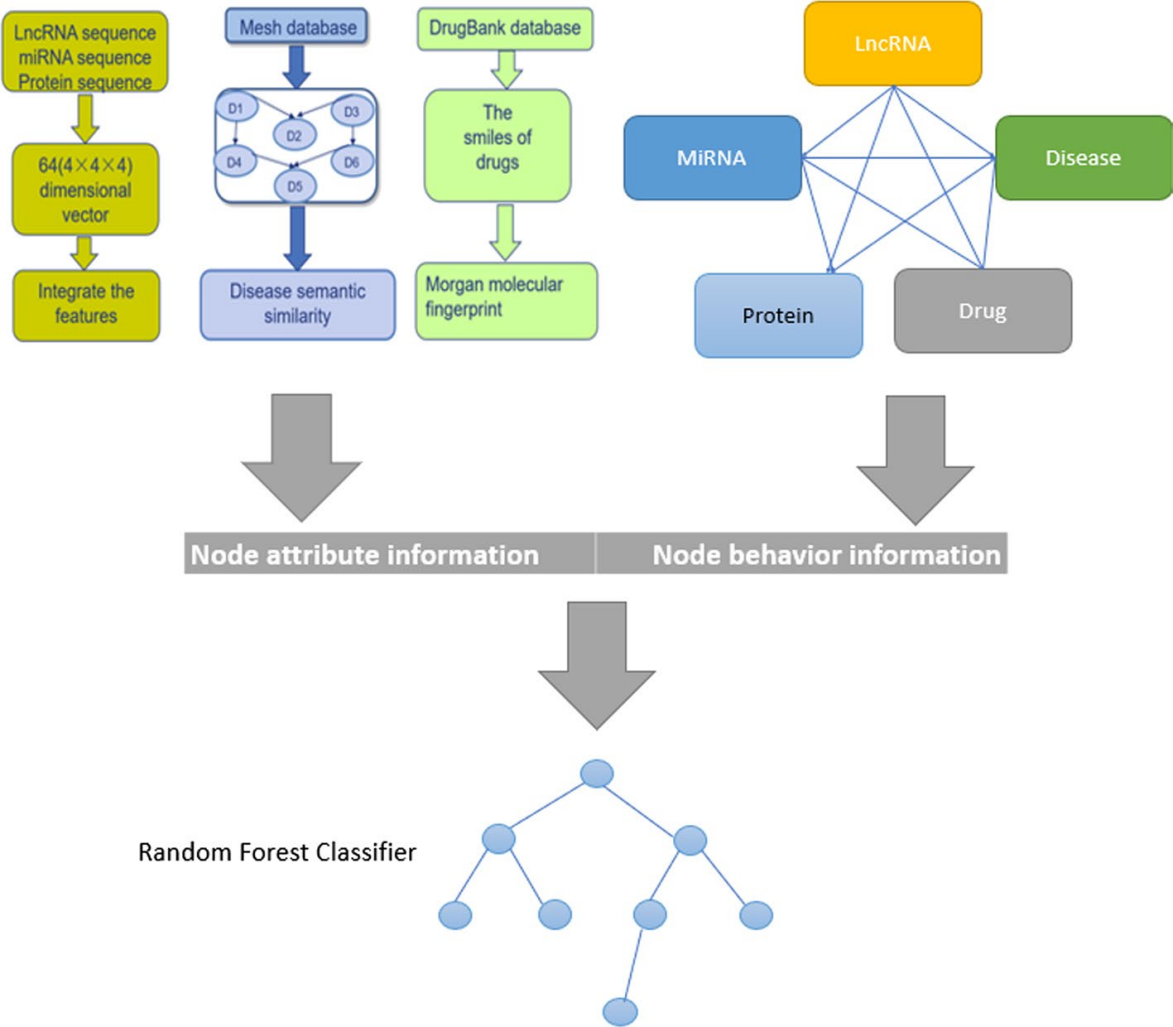
The flowchart of DWLMI

The framework of the proposed DWLMI model for lncRNA-miRNA association prediction is given in Fig. 3, DWLMI model consists of the following main stages:

- **Step 1** We collected our data from multiple databases, after a series of dealing with our data, such as de-redundancy, simplification, and deletion of the irrelevant items, we collect five biological objects, besides that, the associations among them were also collected.
- **Step 2** We construct a global heterogeneous graph to show the relationships among these biological objects, the network embedding method named DeepWalk was used to represent the behavior information of vertices in our molecular network, and the methods of k-mer, Mesh descriptor, fingerprint were used to represent the attribute information of nodes in our network.
- **Step 3** All nodes in our graph can be represented by the attribute information combined with behavior information, the classifier of random forest was used to train our model.

### Heterogeneous associations network

Heterogeneous associations network systematically and holistically collected associations among many types of databases, for example, lncRNASNP2 databases, HMDD databases, all databases were well known and curated experiment-supported evidence. after a series of dealing with the dataset, such as uniform identifiers and elimination of redundancy, 6528 nodes, and 105,546 associations were collected, besides that we can find five biological objects, such as lncRNA, miRNA, drug, protein, and disease. The experiment results are shown in Table 6.

**Table 6:** The associations of different biomolecules in DWLMI

Relationship type	Database	Number of associations
lncrna-miRNA	lncRNASNP2 [40]	8374
Protein-miRNA	miRTarBase [41]	4944
Disease-miRNA	HMDD [42]	16,427
Disease-lncRNA	LncRNADisease [43]	1264
	lncRNASNP2 [40]	
Protein-lncRNA	LncRNA2Target [44]	690
Disease-protein	DisGeNET [45]	25,087
Protein-drug	DrugBank [46]	11,107
Disease-drug	CTD [47]	18,416
Protein–protein	STRING [48]	19,237
Total	N/A	105,546

After aggregating the above database, final statistics are obtained by separately classifying the different nodes as shown in Table 7.

**Table 7:** The amounts of nodes in DWLMI

Node	Amount
LncRNA	769
Disease	2062
Protein	1649
MiRNA	1023
Drug	1025
Total	6528

### Numerical sequence information

The sequences of protein, lncRNA, and miRNA were obtained from LncRNASNP2 [40], NONCODE [49], MiRbase [50], and String [31], respectively, the algorithm of *k*-mer was used to analyze sequence information, the term *k*-mer refers to the substrings of biological sequence with length *K*, such that the sequence GTAA would have four monomers (G, T, A, and A), three 2-mers (GT, TA, AA), two 3-mers (GTA and TAA) and one 4-mer (GTAA). A biological sequence of length *K* can generate *L-K* + 1 k-mers, besides that, the number of possible monomers would have ${n}^{k}$ total possible *k*-mers, for representing the attributes of nodes, the sequence of lncRNA, miRNA, and protein are represented by a 64 (4 × 4 × 4) dimensional vector. With the corresponding 3-mer in sequence, the normalized frequency can be represented by the vector.

### MeSH descriptors and directed acyclic graph

**Fig. 4:**
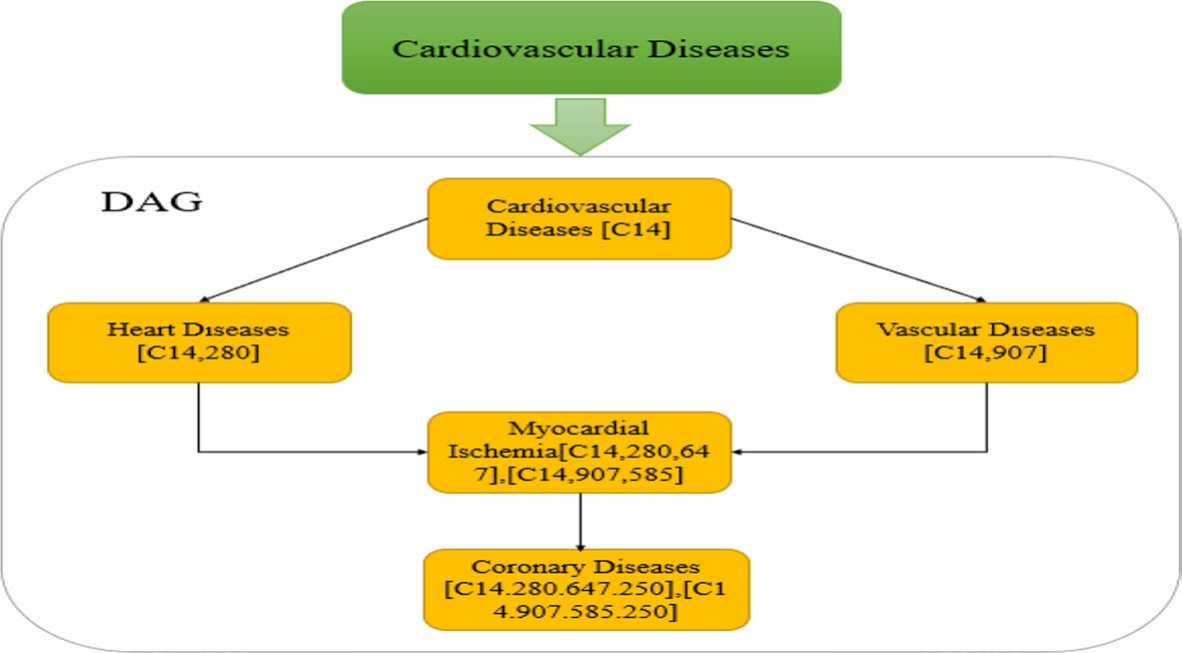
The DAG of coronary disease

National library of medicine creates a widespread searchable controlled vocabulary of MeSH thesaurus, including the headings of the subject and the index and classification used in the life sciences. Due to the structure of the MeSH descriptor hierarchy, the DAG (Directed Acyclic Graph) generated through diseases and MeSH can be used to represent a wide variety of ailments. Details of representing the disease in DAG are: *DAG(D)* = *(D, N(D), E(D)), N(D)* contains all diseases that are represented by points. *E(D)* contains all the associations of nodes in the *DAG(D)* [16]. Figure 4 below shows the DAG of coronary diseases.

The condition is characterized by utilizing DAG included in the Mesh. We define the semantic similarity as follows. Ancestral disease *t* contributes to disease *D*, expressed as the following formula in DAG:

6 $$\left\{ {\begin{array}{ll} {D_{D} \left( t \right) = 1} \hfill & if\quad t = D \hfill \\ {D_{D} \left( t \right) = \max \left\{ {\Delta {*}D1_{D} \left( {t^{{\prime }} } \right)|t^{{\prime }} \in children\,of\,t} \right\}} \hfill & if\quad ift \ne D \hfill \\ \end{array} } \right.$$

The factor of semantic contribution is *∆*. The contribution of node *D* to itself is *I*, and *D* contributed by other nodes will be attenuated due to the total of donations of all diseases that can be obtained in DAG to *D*:

7 $$D_{V1} \left( {\text{D}} \right) = \Sigma_{t \in N\left( D \right)} D_{1D} \left( t \right)$$

Jaccard similarity coefficient is used to calculate the semantic similarity between diseases *i* and *j*:

8 $$S1\left( {i,j} \right) = \frac{{\mathop \sum \nolimits_{t \in N\left( i \right) \cap N\left( j \right)} \left( {D_{1i} \left( t \right) + D_{1j} \left( t \right)} \right)}}{{D_{V1} \left( i \right) + D_{V1} \left( j \right)}}$$

To better understand the process of the semantics calculation between diseases, the systemic lupus erythematosus and acne vulgaris are selected to illustrate the method. First, we construct the directed acyclic graphs of systemic lupus erythematosus and acne vulgaris according to the Mesh descriptors. Second, we calculate the contribution of nodes in the directed acyclic graphs to systemic lupus erythematosus and acne vulgaris. According to the formula above, we can find these two diseases are at the lowest level of their directed acyclic graph, they contribute 1 to themselves, then the parent node contributes 0.5 to themselves, by that analogy, we can calculate the contribution of all nodes in the directed acyclic graph to the systemic lupus erythematosus and acne vulgaris. Finally, we can get the DV values, $DV\left( {{\text{lupus erythematosus}},{\text{ systemic}}} \right) = 2.5$, $DV\left( {\text{acne vulgaris}} \right) = 2.375$, the similarity also was calculated by the formula:${ }Similarity\left( {{\text{systemic lupus erythematosus}},{\text{ acne vulgaris}}} \right) = \frac{0.25 + 0.125}{{2.5 + 2.375}} = 0.0769$.

### Drug molecular fingerprint

**Fig. 5:**
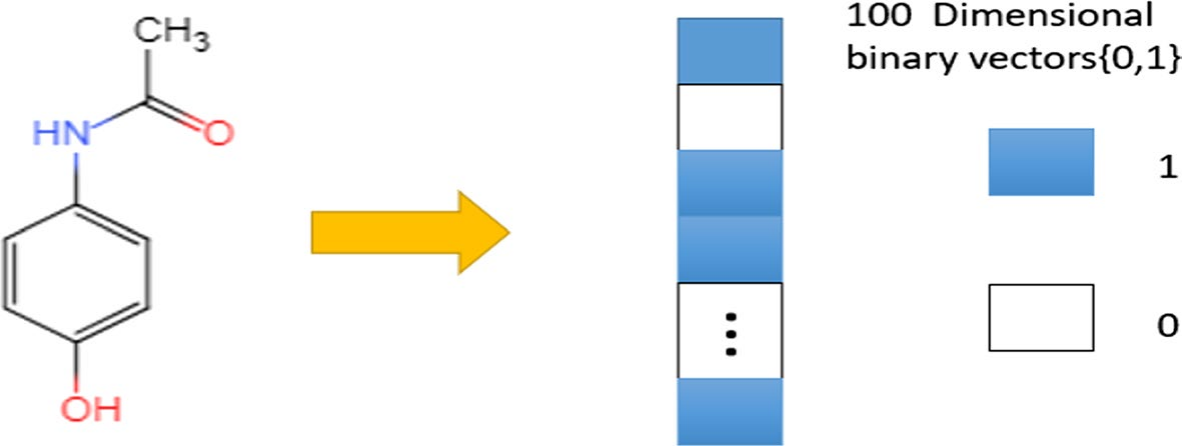
The fingerprint structure of acetaminophen

Molecular fingerprints are a way to show the structure of a molecule by using binary digits to represent the special infrastructures in the molecule. The fingerprint structure of acetaminophen is shown in Fig. 5. The DrugBank database includes detailed drug and inclusive drug target information, such as the chemical data and structure information of drug targets. In our experiment, the similarity of drugs was obtained from DrugBank, then, a cheminformatics toolkit named RDKIT was used to transform the similarity of drugs to Morgan Fingerprint to show the feature of the drug, it is worth noting that RDKIT is binding for python, so the experiment is carried out under python environment.

### Stacked autoencoder

Autoencoder can learn the features of input data. By learning new features, original input data can be reconstructed. The data of the output layer can be reconstructed by understanding the hidden layer.

**Fig. 6:**
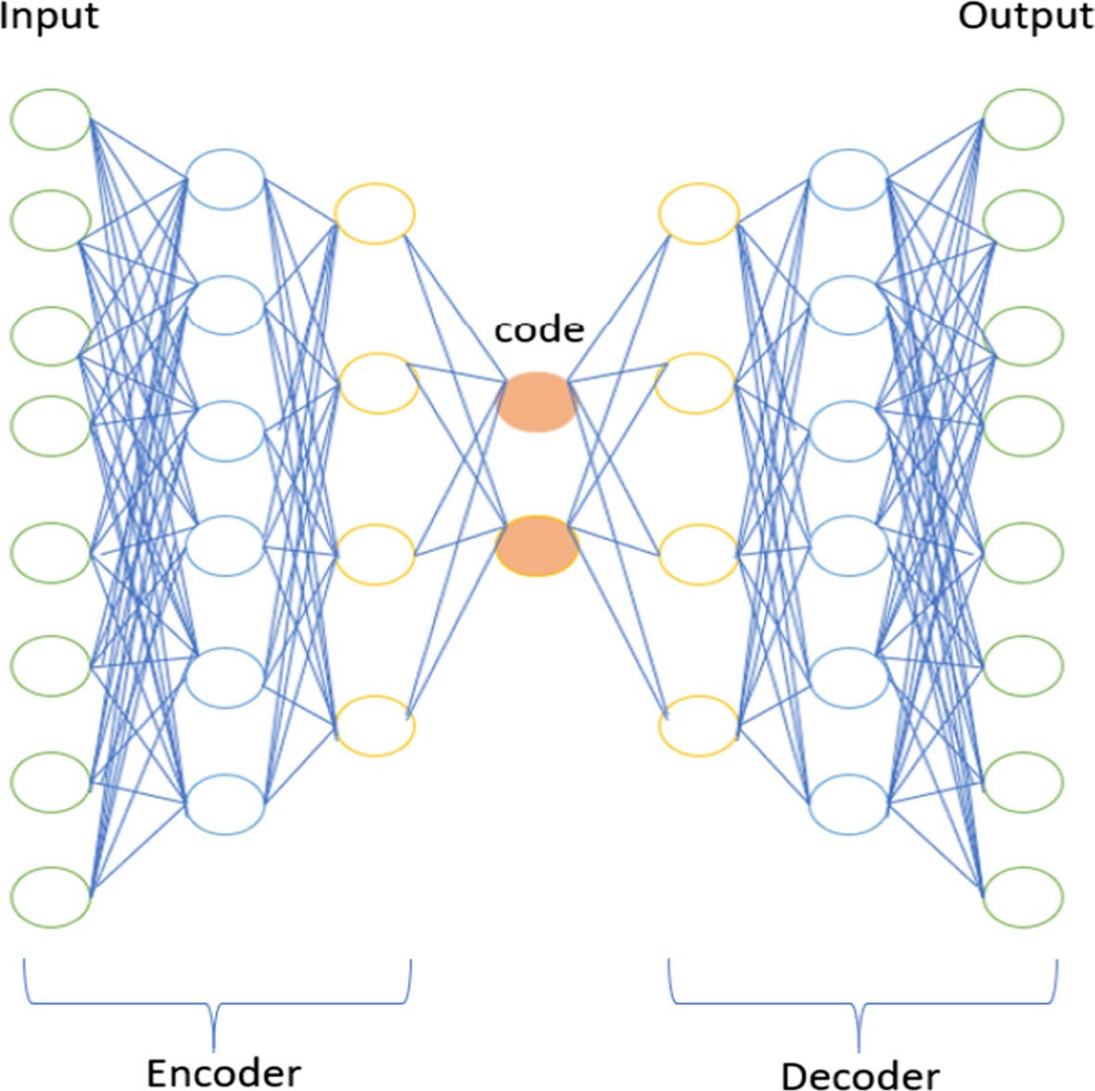
The structure of stacked autoencoder

The stacked autoencoder is a stack of an autoencoder and can be used to improve accuracy by normalizing attribution information to a uniform dimension. The basic structure of the Stacked Autoencoder is shown in Fig. 6.

The encoder and the decoder constitute SAE. The encoder can turn the input data into the corresponding representation *h*, and hidden representation *h* can be reconstructed as an approximation *x* by the decoder.

9 $$h = f\left( x \right): = Sf\left( {wx + p} \right)$$

10 $$y = g\left( h \right): = Sg\left( {w^{{\prime }} x + q} \right)$$

ReLU function as the activation function:

11 $$Sf\left( t \right) = Sg\left( t \right) = \max (0,wt + b)$$

### Learning node representation by DeepWalk

In our experiment, the heterogeneous network is constructed to describe the association systematically. The intrinsic attributes can be represented by their features. The relationship with other nodes of each node can be represented by a network embedding algorithm. In our method, nine kinds of molecular associations, such as lncRNA-miRNA associations, miRNA-disease associations, protein–protein associations were collected from multiple databases, then, we combined all associations to construct a heterogeneous network to represent their associations, then, DeepWalk was selected as the algorithm of network embedding to obtain the behavior information of the molecular network. DeepWalk is scalable, so it can deal with large representations for graphs, besides that, for sparse data, DeepWalk outperforms other methods, utilization of DeepWork can make our network easier to generalize in statistical learning. That is the reason why we choose DeepWalk as the algorithm of network embedding. Based on the idea of collaborative filtering, the heterogeneous network can use DeepWalk to transform the relationships between nodes and other nodes as a vector through network embedding.

In the algorithm of DeepWalk, the graphs can be used as input, we can obtain latent representation from output to generalize a useful model to process a particular language by DeepWalk [51], then local information was used to learn latent phrases of vertices in a network as the equivalent of sentences were obtained from truncated random walks. Finally, we can get an effective method by truncated random walks and language models. Table 8 below describes the whole algorithm in detail.

**Table 8:**
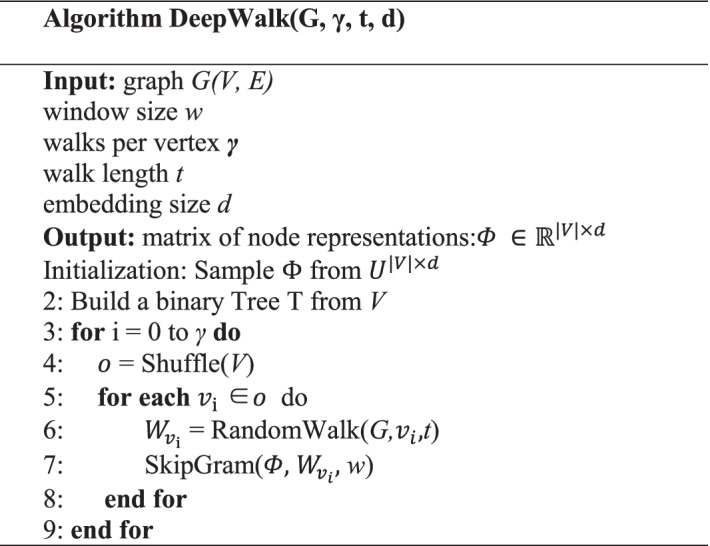
The DeepWalk overall algorithm

Let *G* = (*V*, *E*), where *V* represents the nodes in our molecular network, *E* are their associations, $E \in (V \times V)$, *and* $\Phi \in {\mathbb{R}}^{\left| V \right| \times d}$, where *d* is the dimension of each attribute vector in the feature space. DeepWalk consists of two parts: an updater and a random walk generator. First, the random walk estimates the probability of the next node:

12 $$p_{r} (v_{{\text{i}}} |(v_{1} ,v, \ldots ,v_{{{\text{i}} - 1}} ))$$

Then, a mapping function will be used to show the hidden social representation between nodes. A mapping function $\Phi :v \in V \to {\mathbb{R}}^{\left| V \right| \times d}$.

13 $$p_{r} (v_{{\text{i}}} |(\Phi (v_{1} ),\Phi (v_{2} ), \ldots \Phi (v_{{{\text{i}} - 1}} )))$$

Finally, the Skip Gram module is used to optimize:

14 $${\text{Minimize }} - \log p_{r} \left( {\left\{ {v_{{{\text{i}} - {\text{w}}}} , \ldots ,v_{{{\text{i}} + {\text{w}}}} } \right\}\backslash v_{{\text{i}}} |\varphi (v_{{\text{i}}} )} \right)$$

## Data Availability

The datasets used and/or analyzed during the current study are available from the author on reasonable requests.

## Abbreviations

LncRNA: Long non-coding RNA
MiRNA: Micro RNA
DAG: Directed acyclic graph
ROC: Receiver operating characteristic curve
AUC: The areas under the receiver operating characteristic curve
AUPR: The areas under the precision-recall curve
PR: Precision-recall curve
MCC: Matthews correlation coefficient
TPR: True positive rate
TNR: True negative rate
